# Dataset from RNAseq analysis of bud differentiation in Ficus carica

**DOI:** 10.1016/j.dib.2023.109418

**Published:** 2023-07-29

**Authors:** Ilaria Marcotuli, Stefania Lucia Giove, Angelica Giancaspro, Agata Gadaleta, Giuseppe Ferrara

**Affiliations:** Department of Soil, Plant and Food Sciences, University of Bari “Aldo Moro”, via G. Amendola 165/A, Bari 70126, Italy

**Keywords:** Brebas, Main crop, Genetics, RNAseq, Fruit production

## Abstract

The presented data regards the transcriptome profiling and differential analysis with RNA-Seq approach with the following goals: de novo transcriptome assembly and genome annotation of Ficus carica and the differential expression analysis of parthenocarpic and non-partenocarpic varieties in order to identify candidate genes for the production of seedless fig. Two fig varieties Dottato and Petrelli and the caprifig were grown at the fig repository at the ‘P. Martucci’ experimental station in Valenzano (Bari) of University of Bari ‘Aldo Moro’.

The data included: RNA-seq data obtained from fruits of parthenocarpic and non-parthenocarpic varieties, gene expression in the different genetic materials; genes up and down regulated.

The data in this article support information presented in the research article “I. Marcotuli, A. Mazzeo, P. Colasuonno, R. Terzano, D. Nigro, C. Porfido, A. Tarantino, R. Aiese Cigliano, W. Sanseverino, A. Gadaleta, G. Ferrara, Fruit Development in Ficus carica L.: Morphological and Genetic Approaches to Fig Buds for an Evolution From Monoecy Toward Dioecy. Front. Plant Sci.(2020) 11:1208. doi: 10.3389/fpls.2020.01208

**Specifications Table**

**Table:** 

Subject	*Biological sciences*
Specific subject area	*Genetics: General* *Plant Science: General*
Type of data	Tables Figures FASTA/FASTAQ files
How the data were acquired	Samples used for the analysis were collected from two fig varieties, Dottato and Petrelli, and one profig at two different timepoints, April and July, obtaining fiorone and fico for each genotype, respectively. Total RNA was extracted according to the RNeasy Plant Mini Kit (QIAGEN®) instructions for the two stages with three different biological replicas, and for each of its three technical replicates. RNA quality and quantity were assessed by Nanodrop 2000 (Thermo Scientific, USA) and checked on 1.5% agarose gel [1], while RNA integrity was evaluated with Bioanalyzer 2100 and TapeStation 4200. Samples with a RIN higher than 8 were used for sequencing. The TruSeq Standard mRNA kit (Illumina USA) was used for library construction, and HiSeq 2000 Illumina system was used for RNA sequencing using a paired-end sequencing technique (2 × 100 bp).
Data format	Raw Analyzed
Description of data collection	Two fig cultivars, Dottato (also known as ‘Kadota’) and Petrelli (San Pedro Type), and a caprifig tree were sampled at two timepoints, April and July in the fig repository at the “P. Martucci” experimental station in Valenzano (Bari) of University of Bari “Aldo Moro” equipped with environmental and soil sensors [2]. For each stage and variety three different biological replicas were used, and for each of its three technical replicates
Data source location	Department of Soil, Plant and Food Sciences, University of Bari “Aldo Moro”, Bari, Italy Via G. Amendola 165/A, 70126 Bari, Italy Locations of the durum wheat field of Valenzano (metropolitan city of Bari –Italy): lat. 41.0438° N, long. 16.8842° E, elevation 85 m above sea level.
Data accessibility	Repository name: NCBI Data identification number: PRJNA623468 Direct URL to data: https://www.ncbi.nlm.nih.gov/biosample?Db=biosample&DbFrom=bioproject&Cmd=Link&LinkName=bioproject_biosample&LinkReadableName=BioSample&ordinalpos=1&IdsFromResult=623468 The genotypes on the NCBI database were reported as fiorone (harvested in April) and fico (harvested in July) [3]. The specification of each genotype (petrelli, Dottato and profig) is reported under the Organism section. Repository name: Mendeley Data identification number: DOI:10.17632/cmf387rt4c.1 Direct URL to data: https://data.mendeley.com/datasets/cmf387rt4c/1 Newly obtained genome (GTF file) and sequences' Gene Ontology annotations of Ficus carica were reported in the repository [4]
Related research article	I. Marcotuli, A. Mazzeo, P. Colasuonno, R. Terzano, D. Nigro, C. Porfido, A. Tarantino, R. Aiese Cigliano, W. Sanseverino, A. Gadaleta, G. Ferrara, Fruit Development in Ficus carica L.: Morphological and Genetic Approaches to Fig Buds for an Evolution From Monoecy Toward Dioecy. Front. Plant Sci.(2020) 11:1208. doi:10.3389/fpls.2020.01208

## Value of the Data

• These data represent an added value on the bud differentiation process knowledge, which can be suitable for understanding what makes a bud developing into a main crop in the current year or enter dormancy and develop into a breba in the following season.
• These data include additional information on genes expressed and up or down regulated during the bud development and differentiation.
• These data can be included in the group of information, which can enrich the lack of info concerning bud differentiation mechanisms behind the different crops.

## 1 Objective

The fruits development of fig is very complex process, since there is a large variability among fig varieties including ones needing pollination and varieties that do not. Additionally, the “main crop” of certain genotypes could be separated in two sub-groups, the main crop, maturing in the period of July-September and the late “main crop”, maturing in autumn and borne on the trees up to December. There are genotypes producing only the main crop that ripe late in the summer season. This “difference” of crops allowed to distinct varieties in uniferous (only main crop), biferous (two crops, breba, and main crop), and triferous (breba, summer, and late main crop) [5,6].

Fig genetic variability can be an interesting resource of genetic variation for breeding and for understanding the parthenocarpic production of figs.

In the present paper was presented the integrated pipeline obtained in order to produce a De novo transcriptome assembly and annotation of Ficus carica.

*Data described in this paper support the published original research article titled:* I. Marcotuli, A. Mazzeo, P. Colasuonno, R. Terzano, D. Nigro, C. Porfido, A. Tarantino, R. Aiese Cigliano, W. Sanseverino, A. Gadaleta, G. Ferrara, Fruit Development in Ficus carica L.: Morphological and Genetic Approaches to Fig Buds for an Evolution From Monoecy Toward Dioecy. Front. Plant Sci.(2020) 11:1208. doi:10.3389/fpls.2020.01208.

## 2 Data Description

The goal of the analysis was to improve and complete the already available F. carica annotation data by integrating different sources of information.

The repository database NCBI contains six folders, each one containing the raw sequence reads of Dottato, Petrelli and caprifig at the two timepoints. The entries are named using the abbreviation of the type of bud, the name of the genotype and the month of sample harvesting as following: FDA (Fiorone Dottato April), FDLb (Fico Dottato July), FPA (Fiorone Petrelli April), FPL (Fico Petrelli July), PRA (profig caprifig April) and MLb (mammone Caprifig July).

The Mendeley Data repository database contains two files, one with the newly obtained genome annotation (GTF file) and a second one with the sequences' Gene Ontology annotation in standard format file

## 3 Experimental Design, Materials and Methods

### 3.1 Genome Annotation and RNA-seq Analysis

**Fig. 1:**
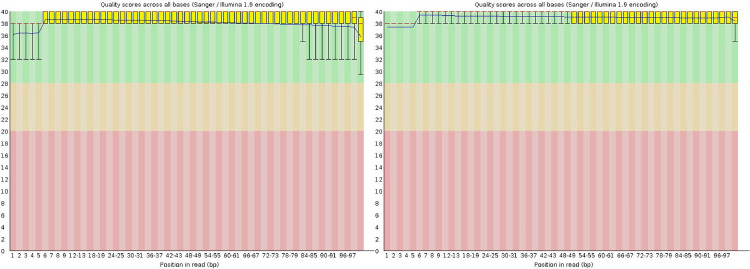
Summary of the quality of the sequenced bases in fico and fiorone of the three genotyoes before (left) and after (right) trimming. The x-axis reports the position along the reads, the y-axis reports the quality as Phred-Like score. The yellow boxes represent the interquartile range of the quality values at each position, the red bar the median, the black line the average quality value. The whiskers of the boxes represent the 10th and the 90th percentile. The scores in the green part of the chart are considered good.

RNA sequencing experiment was performed on 6 samples (three of each genotype at two timepoits). Prior to further analysis, a quality check was performed on the raw sequencing data, removing low quality portions while preserving the longest high-quality part of NGS reads. The minimum length was set to 35 bp and the quality score to 25, using the software BBDuk (Table 1). Quality of the reads was checked before and after the trimming step (Fig. 1).

**Table 1:** Number of reads obtained from RNA sequencing analysis of fig and profig genotypes before and after the quality check (QC).

Sample name	Condition	n° of reads before QC	n° of reads after QC
Dottato	Breba	26,644,282	25,521,050
Dottato	Main crop	29,678,280	28,338,786
Petrelli	Breba	28,391,726	27,099,260
Petrelli	Main crop	30,768,614	29,424,268
Caprifig	Profico	26,489,546	25,396,306
Caprifig	Mammone	25,207,078	24,147,662

## 4 Mapping and Assembly Quality

RNA-seq reads were mapped against the reference genome sequence with STAR (version 2.5.0c) in local mode (Table 2). Then, the reference-guided transcriptome assembly was performed with Trinity (v2.4.0). The number of obtained transcripts was 86,614 and the quality of the assembly was evaluated with different methods:

- Transrate (v1.0.3),
- BUSCO (v3),
- cd-hit-est
- STAR (version 2.5.0c).

**Table 2:** Number and percentage of Uniquely mapped reads, Multi-Mapping reads and Unmapped reads after mapping in fico and fiorone of Dottato, Petrelli and profig.

Sample name	Condition	Uniquely mapped read pairs	Multi-Mapping Read Pairs	Unmapped Reads
Dottato	Breba	91.02%	0.53%	8,45%
Dottato	Main crop	87.08%	0.89%	12,02%
Petrelli	Breba	89.91%	0.51%	9,57%
Petrelli	Main crop	89.26%	0.72%	10,02%
Caprifig	Profico	80.92%	0.82%	18,26%
Caprifig	Mammone	91.80%	0.49%	7,71%

Due to the results obtained, the analysis was carried out using the longest isoforms (read below for more details about the quality results). The quality of the assembly was evaluated again with better quality results. Besides a new quality check was performed with Kallisto, to remove transcripts with no expression. Therefore, after filtering, about 50,866 transcripts were obtained.

## 5 Genome Annotation

Our assembled transcriptome was then merged with a set of transcripts produced by Liceth Solorzano Zambrano, et al. (2017) and used as input for the Maker pipeline.

At the same time an ab initio annotation was performed with Augustus which was also fed to Maker. Four iterations with Maker were performed to improve the Augustus model and finally new gene annotations were obtained. The BUSCO pipeline was then used to check the quality of the raw annotation.

**Fig. 2:**
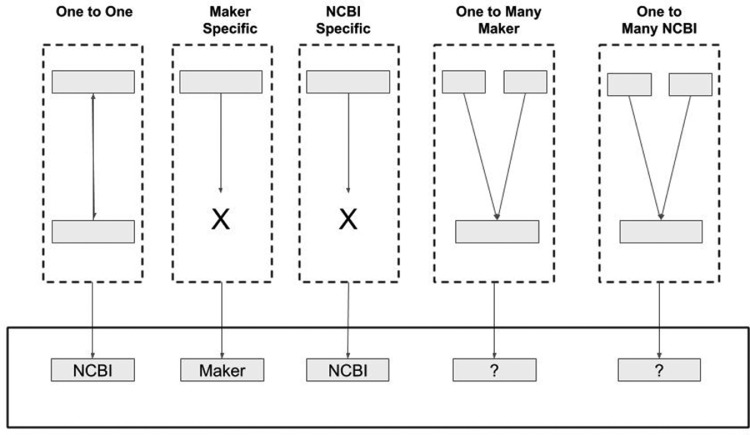
Summary of the rules applied for the gene section in the fig data set using the NCBI and Maker genes against the TrEMBL Plantae and UniProt Plantae database.

A new annotation file (GTF) was obtained with the pipeline which was compared with the “NCBI” annotation by looking at the coordinates of the genes. The following rules were applied (Fig. 2):

• the genes appearing only in the NCBI annotation were always kept. The genes appearing only with the pipeline were analyzed by BLAST against a dataset of plant proteins and only those having a significant match were kept (read below for more details about the BLAST step);
• the genes having a one-to-one match between NCBI and the pipeline, kept the NCBI structure;
• the genes overlapping in a one-to-many way (i.e one NCBI gene matching more Maker genes or vice versa) were analyzed more in depth to understand which was the correct annotation. For this reason, two BLASTP were performed, blasting both the NCBI and the Maker genes against the TrEMBL Plantae and UniProt Plantae database. The results were processed with an in house-script with the following rules:
  ○ if a gene had no BLAST hit, it was removed;
  ○ the coordinates of the BLAST were processed to detect fusion or fragmentation events to keep the correct loci;
  ○ if genes from both the annotation had a hit, then the one with the highest coverage was kept.

The starting NCBI GTF included 36,138 genes, while the new GTF created file counted 35,567 (34,629 in common and 938 new genes).

Therefore, 1509 genes were removed because erroneously annotated based on the new pipeline and supported with Uniprot and TrEMBL database.

Besides, 938 were added as a new gene to the annotation. AHRD (https://github.com/groupschoof/AHRD) was used to assign a description and a Gene Ontology annotation to the sequences.

Finally, the new annotation was evaluated with BUSCO. In order to show the significance of the analysis a new BUSCO Protein analysis was performed taking as reference the Plantae Database.

## 6 Counting

The version 1.4.6-p5 of FeatureCounts software and the new genome annotation were used to analyze gene expression values as raw read counts and to calculate normalized TMM and FPKM values.

## 7 Statistical Analysis

**Fig. 3:**
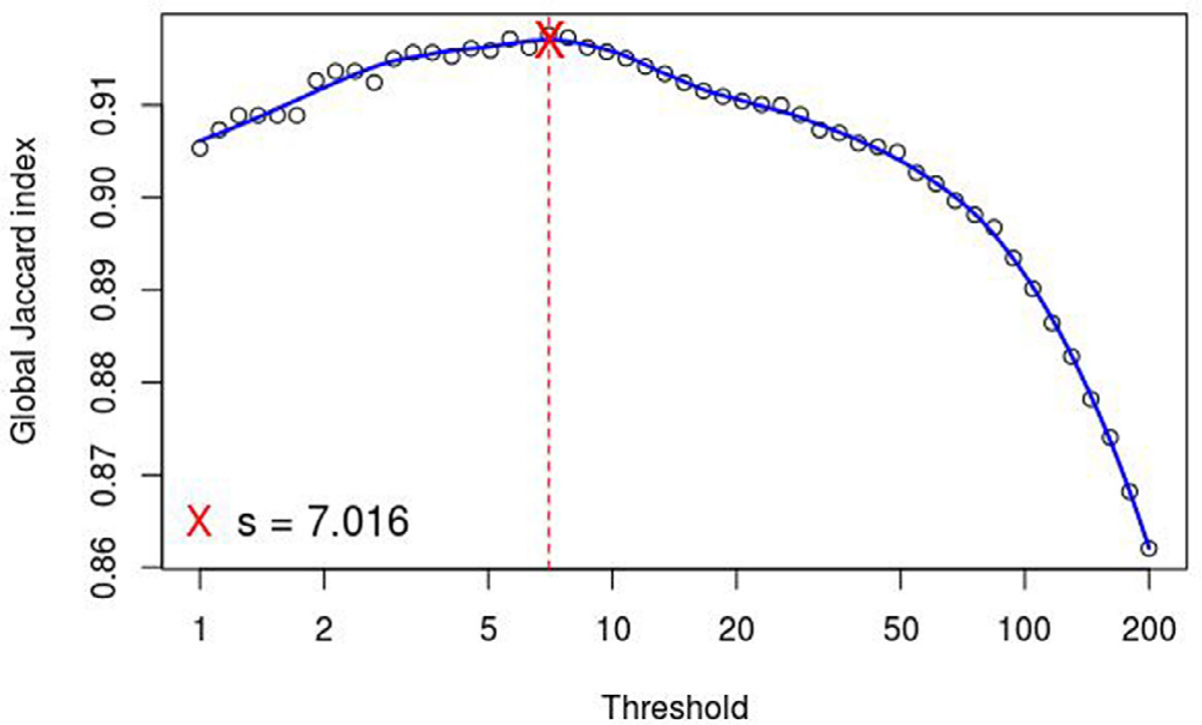
Graphic representation of the HTSFilter analysis result in fico and fiorone of Dottato, Petrelli and profig. The algorithm calculated a Global Jaccard index of similarity between the samples in function of different minimum TMM normalized read counts (s). The graphic shows that for s = 7.016 the replicates have the highest similarity; thus, this value was used as a threshold. All the loci with TMM normalized read counts < s in the samples were removed. This graph is in the file filter.pdf in the folder called 2-DE.

R packages HTSFilter and edgeR software were sued for all the statistical analyses executed., chosen In order to eliminate not expressed genes or ones showing too high variability, the HTSFilter package was applayed implementing a filtering procedure for replicated transcriptome sequencing data based on a Jaccard similarity index. The “Trimmed Means of M-values” (TMM) normalization strategy was also used (Fig. 3). The filter was applied to the different experimental conditions in order to identify and remove genes that appear to generate an uninformative signal.

**Fig. 4:**
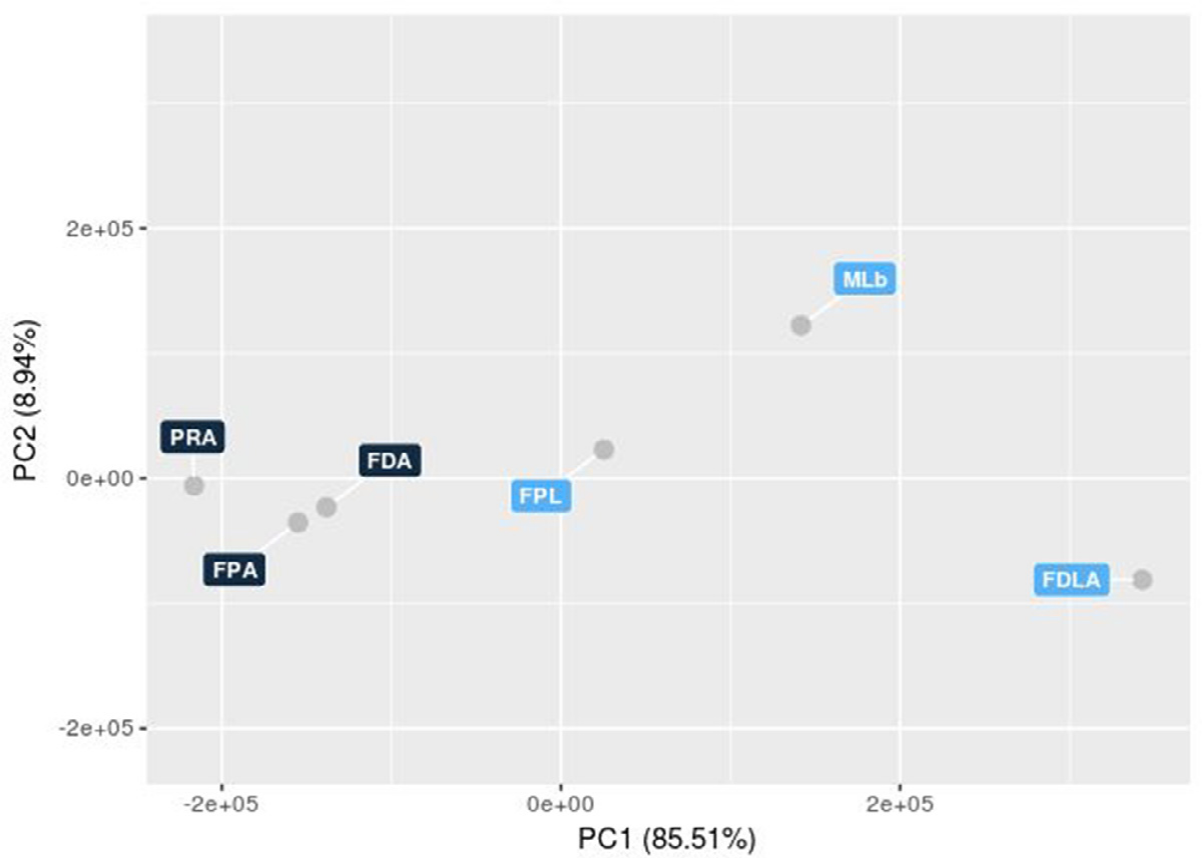
Principal Component Analysis (PCA) conducted on the normalized gene expression in the values of the Dottato, Petrelli and profig samples. X- and Y-axes show the PC1 and PC2, respectively, with the amount of variance explained by each component reported in parenthesis. Each point in the plot represented a sample, dots of the same colors were replicates of a same experimental group.

The overall quality of the experiment was evaluated, on the basis of the similarity between samples, by a Principal Component Analysis (PCA) using the normalized gene expression values as input (Fig. 4).

**Fig. 5:**
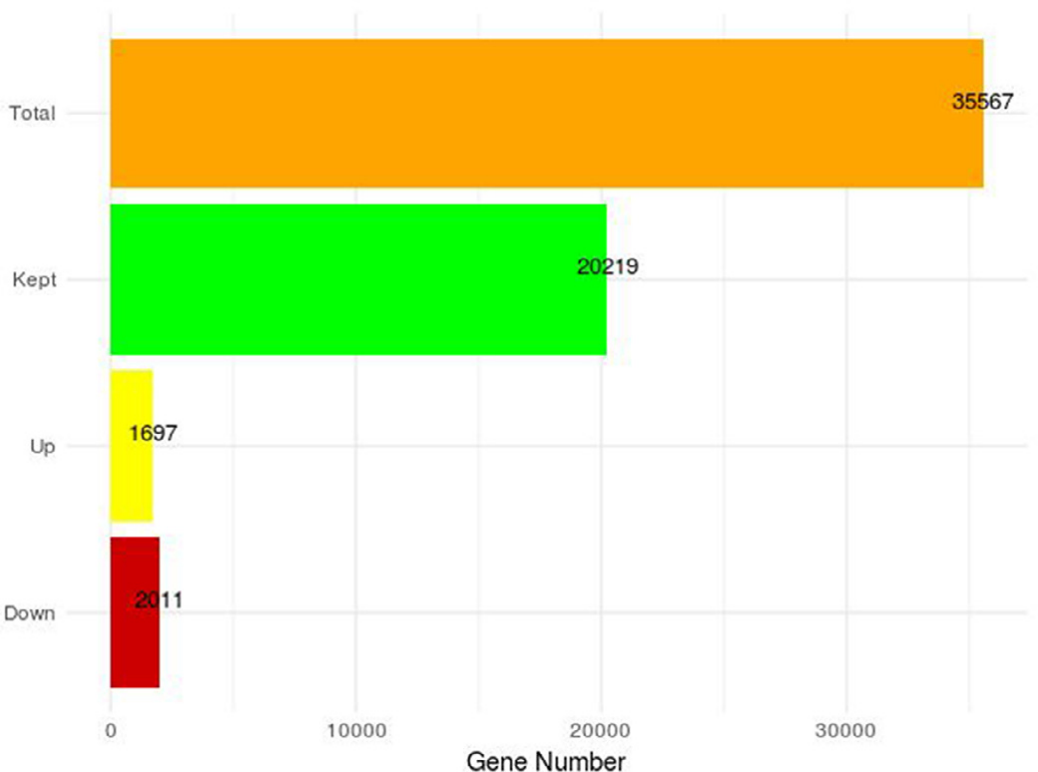
Total number of annotated genes in the reference genome of Dottato (Total), the number of genes that passed the low expression filter (Kept), the total number of differentially expressed up-regulated genes (UP) the total number of differentially expressed down-regulated genes (Down) and across the comparison.

Differential expression analysis was achieved comparing the breba group against the main crop group used as reference allowing the detection of 3708 genes differentially expressed (1697 of them up-regulated and 2011 of them down-regulated) (Fig. 5).

**Fig. 6:**
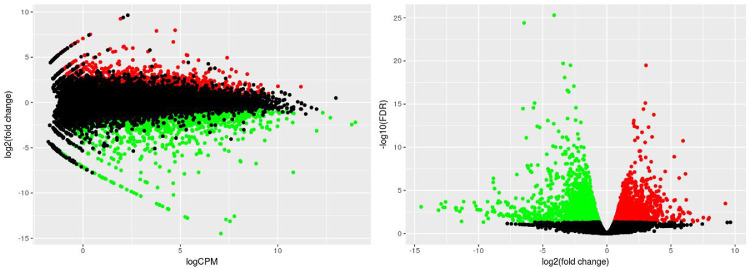
MA plot (On the left) and Volcano plot (on the right) on the genes identified in the fig dataset. Black dots represent the genes that are not significantly differentially expressed, while red and green dots are the genes that are significantly UP- and DOWN-regulated, respectively.

MA and Volcano plot were also made (Fig. 6). On one hand, the MA plot displayed the relationship between the average expression value (on the X-axis) and the fold change (Y-axis) for each gene analysed. The distribution of the dots in the MA-plot were suitable to check if the differentially expressed genes were equally distributed across the different ranges of expression values and the relationship with the fold-change. On the other hand, the Volcano plot showed the relationship between the fold-change (on the X-axis) and the significance of the differential expression test (Y-axis) for each gene in the genome (Fig. 6 right). The distribution of the dots in the Volcano plot was used to detect the range of fold-changes associated with a stronger or a weaker significance of differential expression.

## 8 Gene Ontology Enrichment Analysis

GOEA, Gene Ontology Enrichment Analysis was performed to identify the most enriched Gene Ontology (GO) categories across the down- and up-regulated genes only for the significantly differentially expressed genes.

## Ethics Statements

The work does not involve human subjects, animal experiments, or any data collected from social media platforms.

## CRediT authorship contribution statement

**Ilaria Marcotuli:** Methodology, Software, Data curation, Writing – original draft, Writing – review & editing, Funding acquisition. **Stefania Lucia Giove:** Formal analysis. **Angelica Giancaspro:** Formal analysis. **Agata Gadaleta:** Conceptualization, Validation, Writing – original draft, Writing – review & editing. **Giuseppe Ferrara:** Conceptualization, Validation, Writing – original draft, Writing – review & editing, Funding acquisition.

## Data Availability

PRJNA623468 (Original data) (NCBI). Ficus carica annotation (Original data) (Mendeley Data).
